# Three Arterial Ruptures in a Patient with Neurofibromatosis Type 1

**DOI:** 10.3400/avd.cr.20-00174

**Published:** 2021-06-25

**Authors:** Shotaro Higa, Takaaki Nagano, Junji Ito, Akino Uejo, Moriyasu Nakaema, Yuya Kise, Satoshi Yamashiro

**Affiliations:** 1 Department of Thoracic and Cardiovascular Surgery, Graduate School of Medicine, University of the Ryukyus, Nishihara-cho, Okinawa, Japan; 2 Department of Radiology, Graduate School of Medicine, University of the Ryukyus, Nishihara-cho, Okinawa, Japan

**Keywords:** neurofibromatosis type 1, renal artery, endovascular treatment

## Abstract

Neurofibromatosis type 1 (NF-1) is a rare disease known to cause vascular fragility. A case of a 59-year-old man with NF who had ruptures in three different arteries within a month is presented. The first rupture occurred in the right renal artery and was treated using a stent graft and embolization coils. The second and third ruptures occurred in an artery that had been compressed by a hematoma formed during the first bleed; both were embolized. In patients with NF-1, blood vessel fragility must be considered in treatment selection, especially when performing surgery or other invasive procedures near the great vessels.

## Introduction

Neurofibromatosis (NF) is an autosomal dominant disorder with an estimated prevalence of 1 per 3000–4000 individuals.^1)^ Neurofibromatosis type I (NF-1), its most common subtype, also known as von Recklinghausen’s disease, involves a genetic modification on the long arm of chromosome 17. NF-1 is characterized by the formation of benign neurofibromas, cutaneous café-au-lait spots, and iris hamartomas.^2)^ Other notable features include learning disabilities and skeletal abnormalities. This vascular disease is further characterized by aneurysmal and vascular lesions and vessel wall fragility. Although repeated aneurysm rupture due to vessel fragility has been reported,^3)^ there are no reports of repeated aneurysm rupture within a short period of time. A case involving rupture of three different arteries within a month in a patient with NF-1 is herein presented. The patient in this case agreed to the publication of the case details and images in this report.

## Case Report

**Figure 1:**
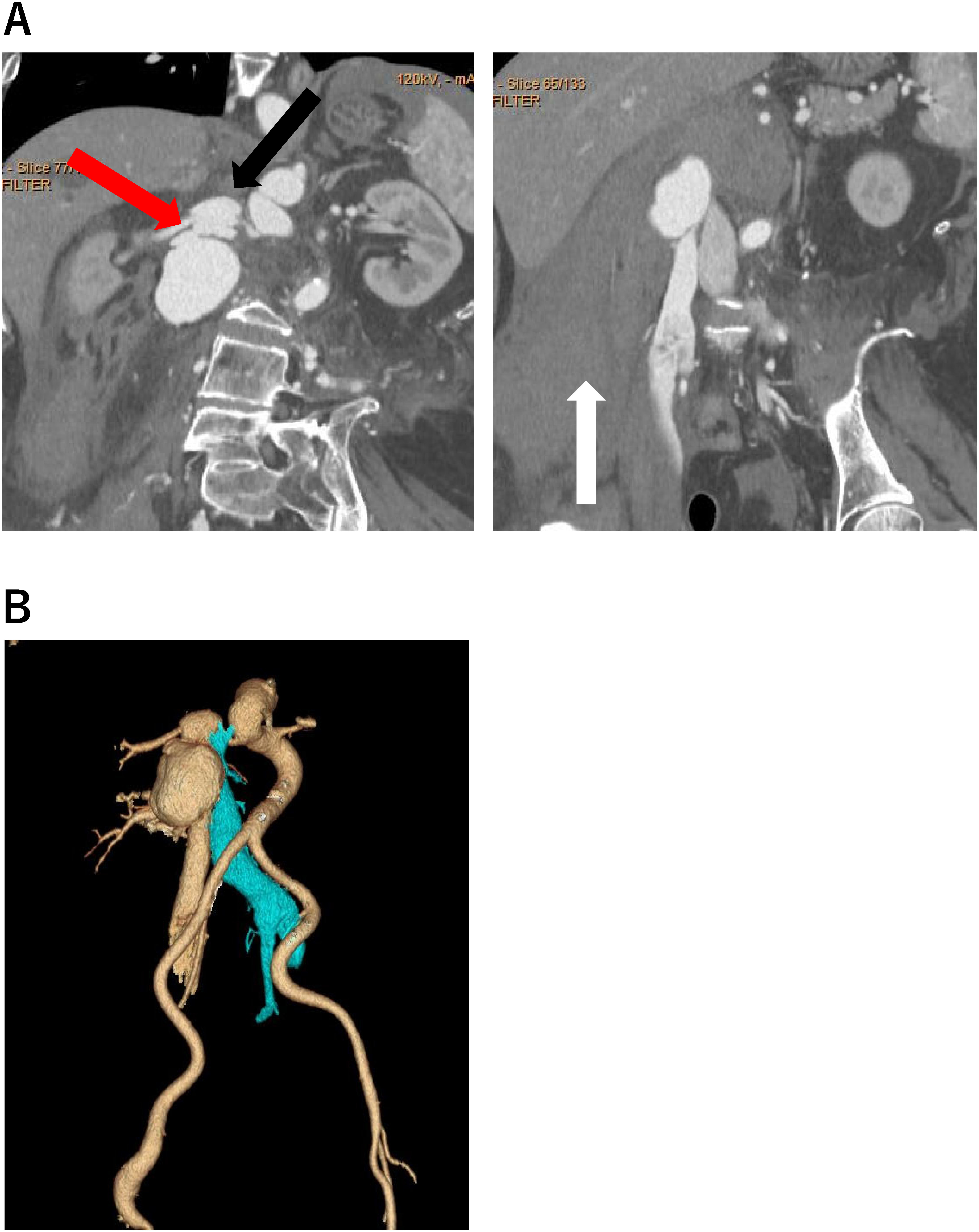
Fig. 1 Preoperative contrast-enhanced computed tomography (CT). (**A**) Enhanced CT showing a right renal artery aneurysm (black arrow), fistula formation to the inferior vena cava (red arrow), and a large hematoma in the right retroperitoneal space (white arrow). (**B**) A three-dimensional CT image.

**Figure 2:**
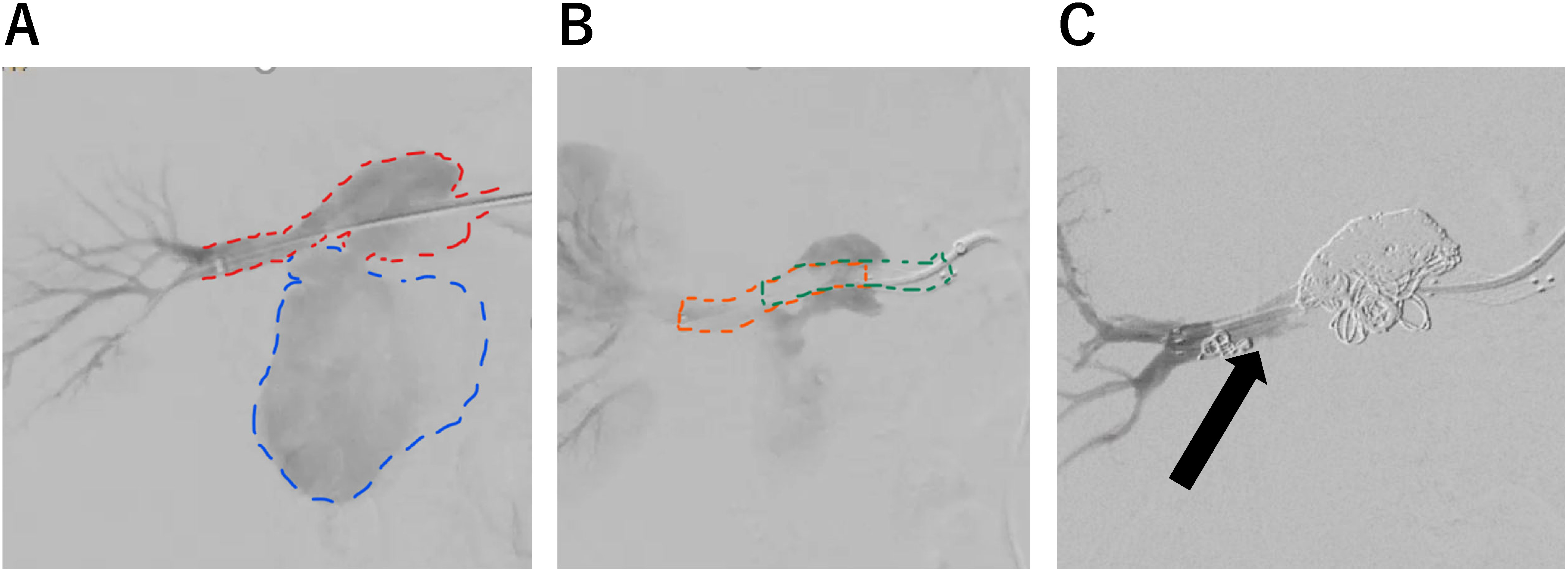
Fig. 2 Angiography. (**A**) Preoperative angiography showing the right renal artery aneurysm (red dotted line) and arteriovenous fistula to the inferior vena cava (blue dotted line). (**B**) Intraoperative angiography shows the residual leak (Viabahn: orange and green dotted lines). (**C**) A postoperative angiogram shows the slight residual leakage between Viabahn and the right renal artery after the additional coil embolization (black arrow).

A 59-year-old man previously diagnosed with NF-1 experienced sudden abdominal pain and vomiting; he was then immediately transported by ambulance to our hospital. Upon arrival in the emergency room, he was conscious and had a blood pressure of 84/68 mmHg, a pulse rate of 132 beats/min, and a hemoglobin level of 11.5 g/dL. Physical examination revealed right lower abdominal pain, café-au-lait spots over his trunk, and neurofibromas. Contrast-enhanced computed tomography (CT) showed a right renal artery aneurysm (21×34 mm), a varicose venous aneurysm (40×41 mm), fistula formation to the inferior vena cava, a large hematoma in the right retroperitoneal space (**Fig. 1**), and an ectatic right femoral artery. On an emergency angiogram, the right renal aneurysm and arteriovenous fistula were also observed through the left femoral artery (**Fig. 2**).

Preoperative CT revealed that the diameters of the right renal artery proximal and distal to the aneurysm were 3 and 4.3 mm respectively. Therefore, two 5 mm×5 cm Viabahn stent grafts (W. L. Gore & Associates, Newark, DE, USA) were placed in the right renal artery using 6 Fr Ansel guiding sheath (Cook Medical, Bloomington, IN, USA) through the left femoral artery. Leakage occurred although post-dilatation was performed using a Sterling Balloon Dilatation Catheter (Boston Scientific, Marlboro, MA, USA). Consequently, embolization using Interlock coils (Boston Scientific, Marlboro, MA, USA) and Nester coils (Cook Medical, Bloomington, IN, USA) was performed to fill the renal artery aneurysm and the gap between the renal artery and the stent graft. Although an angiogram showed slight residual leakage from the renal artery aneurysm, the procedure was finished because complete thrombosis of the aneurysm was expected.

After the procedures, the abdominal pain of the patient resolved, his hemoglobin level was 11.0 g/dL, and 6 U of red blood cells were transfused. As CT revealed, the varicose venous aneurysm was smaller in diameter 3 days after the operation and contained a large number of thrombi. Carbazochrome sodium sulfonate hydrate (100 mg/day) and tranexamic acid (2000 mg) were intravenously administered on postoperative days 1–4. Aspirin (100 mg) was administered to prevent thrombus formation in the stent graft beginning on postoperative day 3.

**Figure 3:**
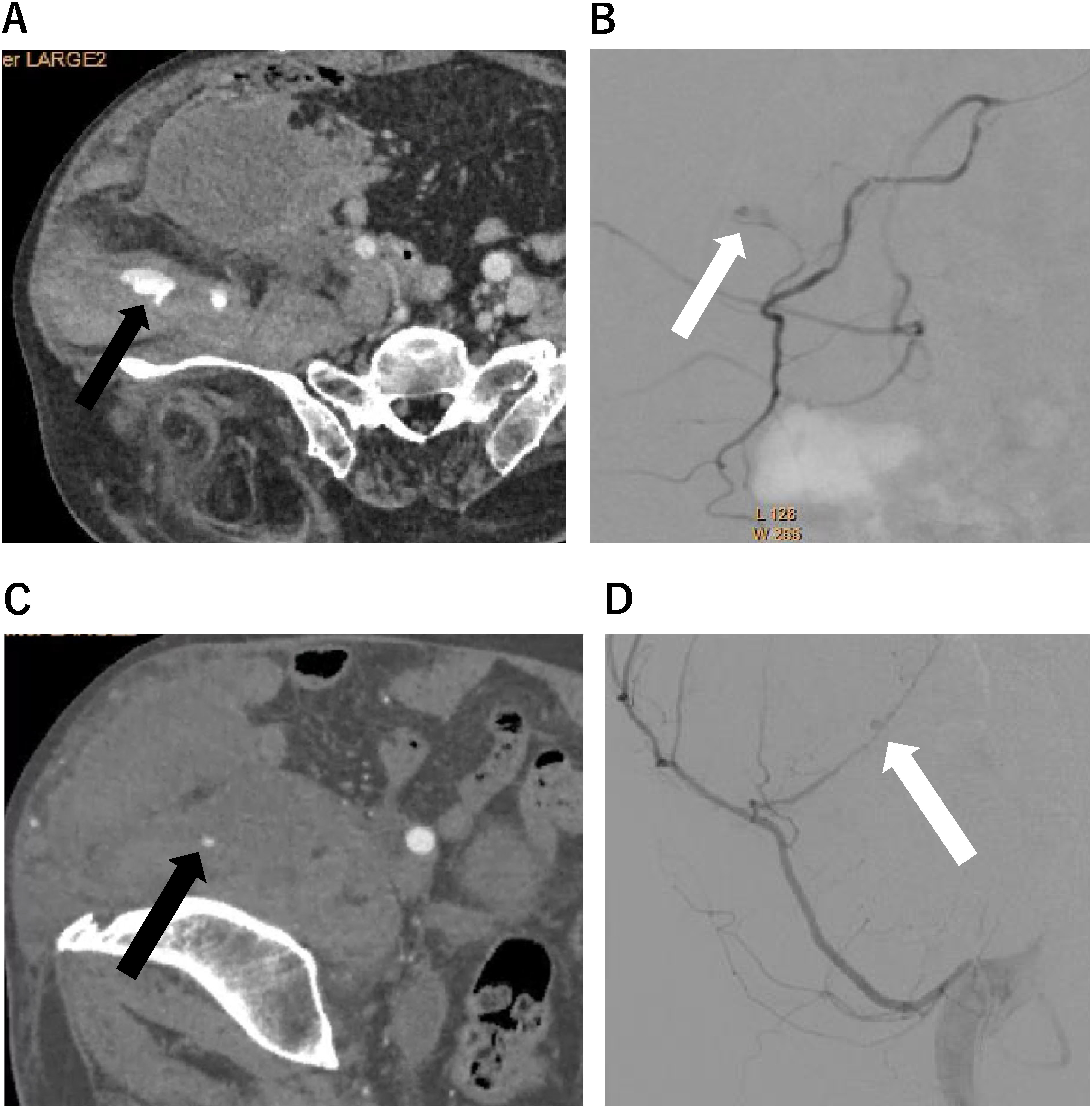
Fig. 3 Contrast-enhanced computed tomography (CT) and angiography on postoperative days 11 and 24. (**A**) Contrast-enhanced CT on postoperative day 11 shows a retroperitoneal hematoma and extravasation from the lumbar artery (black arrow). (**B**) An emergency angiogram shows extravasation from the lumbar artery (white arrow). (**C**) Contrast-enhanced CT at postoperative day 24 shows a pseudoaneurysm at the branch of the deep iliac artery (black arrow). (**D**) An emergency angiogram shows a pseudoaneurysm at the branch of the deep iliac artery (white arrow).

On postoperative day 11, the patient experienced another episode of abdominal pain, and his hemoglobin level was 9.7 g/dL. Imaging revealed a retroperitoneal hematoma (contrast-enhanced CT, **Fig. 3**) and extravasation from the L4 lumbar artery (contrast-enhanced CT, **Fig. 3**; emergency angiogram through the left femoral artery). This artery was selected using a 4 Fr JR2.5 catheter (Medikit, Tokyo, Japan). Using a 2.7 Fr Carnelian HF catheter (Tokai Medical Products, Aichi, Japan), a 1.8 Fr Carnelian MARVEL catheter (Tokai Medical Products) was coaxially inserted through the 4 Fr catheter. This was followed by embolization with a 1 : 2 ratio of Histoacryl (B. Braun Surgical, Barcelona, Spain) and Lipiodol (Guerbet, Paris, France) to block the responsible blood vessel (**Fig. 3**).

The patient experienced the third episode of abdominal pain 13 days later. CT revealed a pseudoaneurysm at the branch of the deep iliac circumflex artery (**Fig. 3**). An emergency angiogram was performed, followed by embolization (1 : 4 ratio of Histoacryl and Lipiodol) to block the responsible blood vessel. After the procedure, the abdominal pain of the patient resolved (**Fig. 3**). The clinical course of the patient has been good 6 months since the first surgery, with no recurrence of abdominal pain. The patient in this report underwent CT 1 month, 3 months, and 6 months later; however, no recurrence of symptoms was observed.

## Discussion

Vasculopathy in patients with NF-1 is rare, with a reported incidence of 2.3%–3.6%.^2,4)^ However, because most NF-1 lesions are clinically silent, this is probably an underestimate. The spectrum of vasculopathy in NF-1 includes aneurysms, stenosis, and medium- and large-vessel arteriovenous malformations. The common sites of aneurysm formation include the aorta and renal, mesenteric, carotid, and vertebral arteries.^5,6)^ Younger patients are more likely to experience aortic, renal, mesenteric, or carotid-vertebral stenoses and aneurysms than older patients.^5)^

Maintaining hemostasis during artificial blood vessel replacement in patients with NF-1 with vascular lesions is difficult.^5)^ Many endovascular treatments include stent grafts or coils.^7,8)^ In the report by Bargiela et al., only 4/66 (6%) patients died after endovascular treatment for NF vascular lesions, despite ruptures in 42 (63.6%) patients. Since the incidence of other complications is not high, endovascular treatment for NF is relatively safe, even for patients with unstable hemodynamics.^8)^ Leier et al. proposed two potential causes of arterial ruptures: the weakening of the vessel wall due to neurofibromatous invasion of the tunica media and the weakening of an arterial segment due to compression of the large-artery vasa vasorum by the neurofibromatous tissue.^9)^

In 2014, the Japan Medical Safety Research Organization issued a warning regarding the need to carefully consider the risk of vascular injury when performing surgery or other invasive procedures near the great vessels in patients with NF-1 owing to their vascular fragility.^10)^ Hence, endovascular treatment rather than nephrectomy or open surgical resection and reconstruction of the right renal artery was chosen as our treatment procedure. Although an arteriovenous fistula was encountered after the first rupture, two assumptions were made: the aneurysm would disappear after the placement of the stent grafts, and the shunting of blood flow through the arteriovenous fistula would be controlled. Unfortunately, the leak remained and required coil embolization. Owing to the residual leak and the surrounding hematoma, the blood vessel measurement may have been underestimated. Given the need for an emergency operation, Viabahn VBX stent grafts were not locally stocked in our hospital and could not be used as there was insufficient time for their preparation. These stent grafts may allow additional dilation in cases where blood vessels have collapsed. Inadvertent migration of coils from the inferior vena cava to the heart should be avoided when embolizing a coil for an arteriovenous fistula. Since, in our case, the arteriovenous fistula was small, a large Interlock coil was first placed on the arterial side as an anchor.

The large size of the irregular saccular aneurysm in an artery that was weakened by NF-1 was the cause of the first rupture, and the quickly following arterial ruptures likely resulted from blood vessel wall fragility and blood vessel stretching caused by the hematoma. A catheter was not inserted into the failed vessel. To prevent the guide wire from entering small blood vessels, it should be visible on the screen. Otherwise, during treatment, careful catheter manipulations are necessary to avoid unnecessary catheter misplacement in blood vessels.

## Conclusion

Multiple endovascular treatments were required for reported arterial ruptures in our patient with NF-1. In such rare cases, careful postoperative observation is necessary owing to vascular fragility in patients with NF-1.

## Additional Note

Written informed consent was obtained from the patient.

## Additional Remarks

This study was presented at the 11th Conference of the German-Japanese Society of Vascular Surgery, Oita, Japan, September 5–7, 2019.

## References

[1] Evans DG, Howard E, Giblin C, et al. Birth incidence and prevalence of tumor-prone syndromes: estimates from a UK family genetic register service. Am J Med Genet A 2010; 152A: 327-32. 20082463 10.1002/ajmg.a.33139

[2] Lin AE, Birch PH, Korf BR, et al. Cardiovascular malformations and other cardiovascular abnormalities in neurofibromatosis 1. Am J Med Genet 2000; 95: 108-17. 11078559 10.1002/1096-8628(20001113)95:2<108::aid-ajmg4>3.0.co;2-0

[3] Misao T, Yoshikawa T, Aoe M, et al. Recurrent rupture of intercostal artery aneurysms in neurofibromatosis type 1. Gen Thorac Cardiovasc Surg 2012; 60: 179-82. 22419191 10.1007/s11748-011-0806-0

[4] Brasfield RD, Das Gupta TK. Von Recklinghausen’s disease: a clinicopathological study. Ann Surg 1972; 175: 86-104. 4621893 10.1097/00000658-197201000-00015 PMC1355163

[5] Oderich GS, Sullivan TM, Bower TC, et al. Vascular abnormalities in patients with neurofibromatosis syndrome type I: clinical spectrum, management, and results. J Vasc Surg 2007; 46: 475-84.e1. 17681709 10.1016/j.jvs.2007.03.055

[6] Delis KT, Gloviczki P. Neurofibromatosis type 1: from presentation and diagnosis to vascular and endovascular therapy. Perspect Vasc Surg Endovasc Ther 2006; 18: 226-37. 17172538 10.1177/1531003506296488

[7] Zhang B, Zou Y, Yang M, et al. Endovascular management of renal artery aneurysms induced by neurofibromatosis type 1: a case report. Medicine (Baltimore) 2017; 96: e8858. 29382000 10.1097/MD.0000000000008858 PMC5708999

[8] Bargiela D, Verkerk MM, Wee I, et al. The endovascular management of neurofibromatosis-associated aneurysms: a systematic review. Eur J Radiol 2018; 100: 66-75. 29496081 10.1016/j.ejrad.2017.12.014

[9] Leier CV, DeWan CJ, Anatasia LF. Fatal hemorrhage as a complication of neurofibromatosis. Vasc Surg 1972; 6: 98-101. 4623800 10.1177/153857447200600208

[10] Japan Medical Safety Research Organization. The risk of the vascular breakdown in von Recklinghausen’s disease. Medical safety information. 2014; 4, https://www.pmda.go.jp/files/000143739.pdf (in Japanese).

